# DNA damage response revisited: the p53 family and its regulators provide endless cancer therapy opportunities

**DOI:** 10.1038/s12276-022-00863-4

**Published:** 2022-10-07

**Authors:** Yasser Abuetabh, H. Helena Wu, Chengsen Chai, Habib Al Yousef, Sujata Persad, Consolato M. Sergi, Roger Leng

**Affiliations:** 1 370 Heritage Medical Research Center, Department of Laboratory Medicine and Pathology, University of Alberta, Edmonton, Alberta T6G 2S2 Canada; 2 Department of Pediatrics, University of Alberta, Edmonton, AB T6G 2E1 Canada; 3 Division of Anatomical Pathology, Children’s Hospital of Eastern Ontario (CHEO), University of Ottawa, Ottawa, ON K1H 8L1 Canada; 4 Present Address: College of Laboratory Medicine, Chongqing Medical University, Chongqing, 400016 China

**Keywords:** Cancer models, DNA damage and repair

## Abstract

Antitumor therapeutic strategies that fundamentally rely on the induction of DNA damage to eradicate and inhibit the growth of cancer cells are integral approaches to cancer therapy. Although DNA-damaging therapies advance the battle with cancer, resistance, and recurrence following treatment are common. Thus, searching for vulnerabilities that facilitate the action of DNA-damaging agents by sensitizing cancer cells is an active research area. Therefore, it is crucial to decipher the detailed molecular events involved in DNA damage responses (DDRs) to DNA-damaging agents in cancer. The tumor suppressor p53 is active at the hub of the DDR. Researchers have identified an increasing number of genes regulated by p53 transcriptional functions that have been shown to be critical direct or indirect mediators of cell fate, cell cycle regulation, and DNA repair. Posttranslational modifications (PTMs) primarily orchestrate and direct the activity of p53 in response to DNA damage. Many molecules mediating PTMs on p53 have been identified. The anticancer potential realized by targeting these molecules has been shown through experiments and clinical trials to sensitize cancer cells to DNA-damaging agents. This review briefly acknowledges the complexity of DDR pathways/networks. We specifically focus on p53 regulators, protein kinases, and E3/E4 ubiquitin ligases and their anticancer potential.

## Cancer therapy: Responses to DNA damage

The tumor suppressor protein p53 is at the center of a network of cellular proteins that coordinate responses to DNA damage, and a deeper understanding of this network could reveal new targets for cancer therapy. Commonly called the ‘guardian of the genome’, p53 helps determine whether cells initiate repairs or self-destruct in response to genomic disruption. Mutations affecting p53 play a critical role in determining tumor response to therapy. Researchers led by Roger Leng at the University of Alberta, Edmonton, Canada, have reviewed the various proteins and pathways that interact with p53, and thus inform its ‘decision-making’ process. The development of drugs that affect p53 directly has proven a huge challenge, but these interacting proteins and pathways could offer therapeutic targets. The authors highlight vulnerabilities that could render tumors more susceptible to radiation or chemotherapy.

## A brief introduction to the DNA damage response

**Fig. 1:**
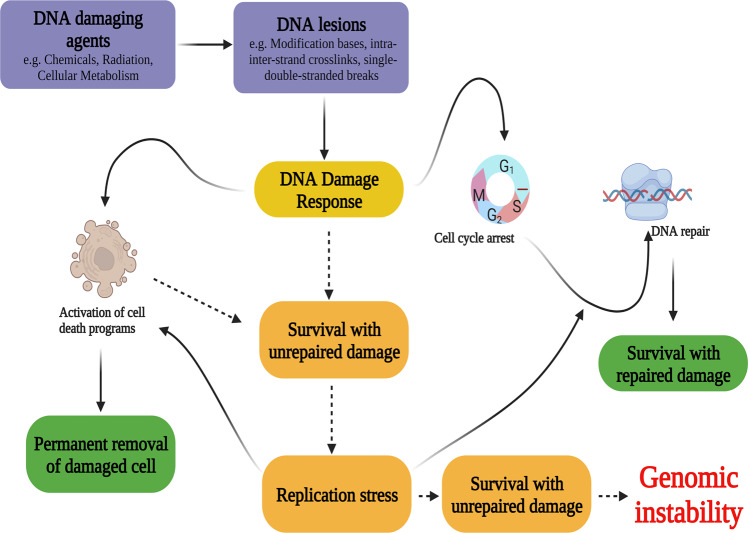
General overview of DNA damage response networks activate by DNA damage. Once cellular DNA damage occurs, the DDR is activated to protect damaged DNA integrity. The cell cycle is paused to provide cells an opportunity to activate DNA repair mechanisms. When the DNA damage is severe, cell death programs are activated. Dashed arrows indicate altered mechanisms. Alterations in DDR networks may lead to the survival of cells with DNA damage, which eventually may lead to one of the main hallmarks of cancer: genomic instability. This figure was created with BioRender.com (granted a license “Academic License Terms”, No. UP246NTDHZ).

Genome integrity is a fundamental factor that guarantees the generating healthy and disease-free daughter cells that constitute healthy homogeneous tissues that are ultimately involved in various biological functions^1^. Hence, genomic instability often leads to diseases, including cancer. It is acknowledged that genomic instability is an established hallmark of cancer formation^2^. Nevertheless, human cells are equipped with precise and sophisticated defense mechanisms that are sufficient and necessary to protect the genome and maintain its integrity against countless internal and external DNA-damaging agents and events^3,4^. These defense mechanisms are collectively named DNA damage response (DDR) pathways. Hence, the DDR can be defined as a complex network of intricate pathways that cooperate to detect, repair, and/or eliminate thousands of DNA lesions in a cell^3^ (Fig. 1). Therefore, the DDR leads to several primary biological outcomes, including cell cycle regression, DNA repair, apoptosis, and senescence. Moreover, alteration in DDR pathways may lead to genomic instability, which is represented by mutation, fusion, deletion, and chromosomal rearrangement or loss. Moreover, an aberrant DDR may lead to various diseases, including neurodegenerative diseases, immunodeficiency, and premature aging. Many details of DNA repair mechanisms and the pathways and molecules involved in the DDR have been revealed in the past few decades.

In general, DDR molecules can be subdivided into (a) DDR sensors, (b) DDR signal transducers, and (c) DDR effectors. For instance, the canonical molecular response to DNA double-strand breaks (DSBs) is highlighted by the recruitment of the MRN (MRE11-RAD50-NBS1) complex to a damage site (formation of radiation-induced foci)^5^. The MRN complex facilitates the recruitment and activation of ataxia telangiectasia mutated (ATM), which, in turn, transduces DDR signals to a set of mediator and effector molecules. In unstressed cells, ATM is a nonactive dimer and monomerizes through autophosphorylation at multiple serine residues, including S1981, in response to DNA damage^6–8^. At a damage site, ATM phosphorylates the histone variant H2AX at S139 (the phosphorylated form is known as γH2AX)^9,10^. γH2AX mediates the recruitment of mediator of DNA damage checkpoint protein 1 (MDC1)^10–12^. Forming a positive feedback loop, MDC1 amplifies the ATM signal by facilitating/recruiting the additional MRN complexes and ATM to the damaged site. Optimal ATM activation requires the recruitment of several other molecules, including p53-binding protein 1 (53BP1), breast cancer type 1 (BRCA1), and the ubiquitin ligases RNF8 and RNF168^13–15^. Ultimately, active ATM phosphorylates/activates several signaling pathways and effectors that mainly modulate cell cycle progression, DNA repair, cell death, cell metabolism, and senescence. On the list of most prolific ATM substrates, tumor suppressor p53 is at the top.

In general, specific DNA damage/lesions are repaired by different DNA repair mechanisms. For instance, DNA single-strand breaks (SSBs) and aberrant base modifications can be repaired by several mechanisms, including base excision repair (BER), nucleotide excision repair (NER), and mismatch repair (MMR). However, DNA double-strand breaks (DSBs) are repaired via different mechanisms, including homologous recombination (HR) and nonhomologous end-joining (NHEJ) repair. All these mechanisms are orchestrated through a variety of different, yet specific, enzymatic cascade reactions (for reviews see refs. ^16–18^). For instance, an oxidized base (such as that formed by the oxidation of deoxyguanosine, generating 8-oxo-deoxyguanosine, which is the most common form of oxidatively damaged DNA) is repaired by BER; via this repair mechanism, the oxidized base is first recognized by members of a distinct enzyme family known as glycosylases, which excise the oxidized base, forming an apurinic/apyrimidinic site (known as an abasic or AP site). An AP site is then excised by AP endonuclease 1 (APE1), which induces a SSB. This nucleotide gap is then filled by the action of different recruited BER proteins, including DNA polymerases, poly [ADP-ribose] polymerase 1 (PARP1) and DNA ligases. The NER mechanism frequently repairs other forms of DNA damage, including pyrimidine dimers (such as T = T), which are commonly caused by UV light exposure^18^.

DSBs are the most threatening types of DNA damage; nevertheless, they can be efficiently repaired by different repair pathways, including the HR and NHEJ repair pathways. A key difference between HR and NHEJ is that HR is recruited exclusively during the S and G2 phases of the cell cycle; in contrast, NHEJ can be activated during all cell cycle phases. HR is generally considered an error-free repair system because a homologous template is the basis of reassembly of the damaged DNA strand. Many proteins that play pivotal roles in initiating and modulating HR have been identified, including the MRN complex, breast cancer susceptibility proteins (BRCA1 and BRCA2), ATM, and ATR (ataxia telangiectasia and rad3-related). In contrast, NHEJ repair is not based on a homologous template. Through NHEJ, the ends of broken DNA are directly rejoined, making it an error-prone repair mechanism. The Ku70/Ku80 complex, DNA-dependent kinases, and X-ray repair cross complementing 4 (XRCC4) are among the most important players in NHEJ.

A comprehensive understanding of DDR pathways has led to the discovery of “synthetic lethality”, which eradicates cancer cells by causing a second deleterious “hit” to a DNA repair mechanism that had been previously damaged. For example, treating BRCA−/− cancers with conventional chemotherapies led to a certain level of resistance because these cancer cells can repair the SSBs induced by treatment. However, using PARP inhibitors (PARPi) with or without chemotherapy led to the accumulation of SSBs, which eventually led to the accumulation of DSBs, which these cancer cells cannot repair due to the lack of the BRCA protein, ultimately resulting in cancer cell death^19,20^. Thus, PARPis have been successfully developed to treat BRCA-deficient patients; however, the clinical efficacy of PARPi has been significantly limited by the relative rarity of BRCA1/2 mutations. There are two major processes of nonhomologous end-joining (NHEJ) repair: classical NHEJ (c-NHEJ) is mediated by the DNA repair factors DNA-PKcs and Ku70/Ku86. PARP1, together with DNA ligase IIIa (Lig3) or DNA ligase I (Lig1), binds a DSB and initiates end-joining via an alternative NHEJ (alt-NHEJ) mechanism^21–25^. Alt-NHEJ is the major DNA repair pathway for pathogenic chromosomal errors^25^. A PARP-DNA lesion generated by PARPi leads to a stalled replication fork and is then repaired predominantly via Alt-NHEJ and HR. Thus, inhibiting Alt-NHEJ in HR-deficient BRCA1-mutant cancers leads to synthetic lethality^25^. BRCA-deficient cells are more sensitive to PARPi than wild-type (wt) BRCA cells^26–30^. PARPi activity is limited due to intrinsic and acquired resistance to these drugs. Thus, treatments are urgently needed to overcome PARPi resistance and enhance PARPi sensitivity.

Factors involved in the DNA damage response and DNA repair machinery are constantly being identified, which will hopefully lead to the development of novel therapeutic strategies to fight cancers.

## The tumor suppressor p53: the prominent guardian of the genome

The tumor suppressor protein 53 gene, *TP53* (encoding p53), has earned the name “guardian of the genome” on the basis of thousands of intensive studies performed over the past few decades, which have implicated its crucial multifunctional role in preserving genomic stability^31^. Indeed, the role played by p53 as a tumor suppressor is fundamentally highlighted by its transcriptional activity/capacity to mediate and regulate genes that directly or indirectly facilitate cell cycle regulation, DNA repair, and cell fate signaling networks; notably, the number of these p53-regulated genes is continuously increasing^32^.

The p53 family members p63 and p73 were identified and characterized several years after p53 discovery^33,34^. Both the p63 and p73 proteins share significant structural and functional similarities. For example, p63 and p73 proteins share conserved structural domains, including DBD (DNA binding domain), which is similar to that in p53. The DBD is highly conserved across all –p53 family members, with the DBD in p63 and p73 showing 65% and 62% homology, respectively, to the DBD in p53. Thus, p63 and p73 control the expression of many genes, similar to p53 regulatory function. Despite their crucial functions as tumor suppressors, p63 and p73, in contrast to p53, are rarely mutated.

Importantly, p63 and p73 exhibit distinct structural domains and different biological functions during development, homeostasis, and diseases. Experiments with p63- and p73-knockout model mice indicated that p73 plays a profound role during embryonic neuronal development, while p63 is important to epithelial development^35–37^. *TP63* and *TP73* (also *TP53*) genes encode a set of different isoforms that are categorized into two groups (TA isoforms and ∆N isoforms) based on the presence or absence of the transactivation domain (TAD), respectively^38–40^. It is generally accepted that TA isoforms are tumor suppressors, while ∆N isoforms are frequently found to possess oncogenic properties. Furthermore, the ∆N isoforms have been found to negatively regulate the levels and activities of the TA isoforms.

**Fig. 2:**
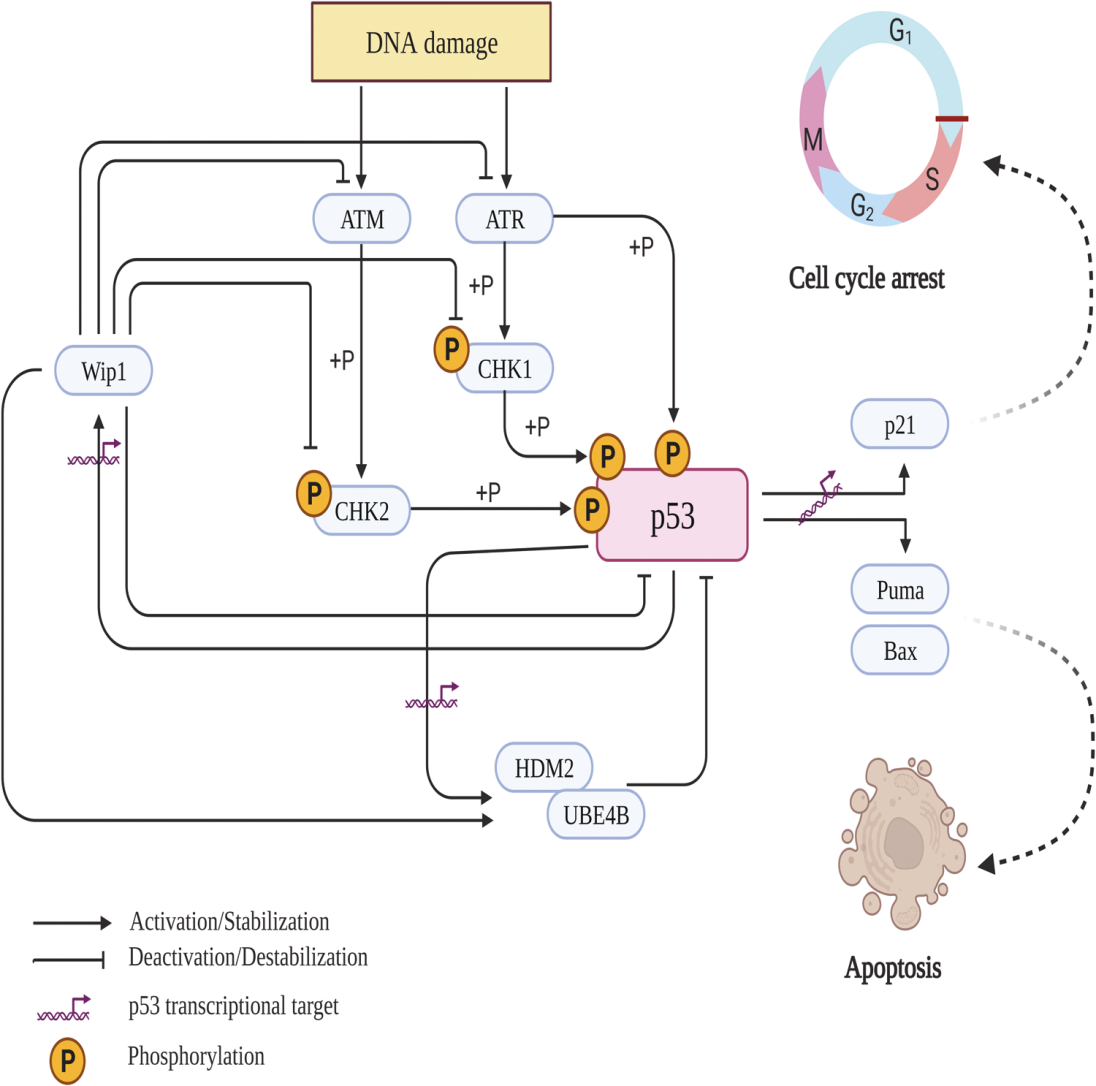
Simplified schematic showing the activation and deactivation of the p53 network in response to a DNA-damaging agent. Under stress conditions, such as ionizing radiation (IR), p53 is rapidly stabilized primarily through phosphorylation mediated by different upstream regulators, such as ATM and ATR. Phosphorylated p53 is stabilized mainly through its disassociation from HDM2 and UBE4B; hence, p53 protein accumulates and is translocated into the nucleus. In the nucleus, p53 aggregates as tetramers, the active forms of p53, and transcriptionally activates or suppresses its targeted genes, including cyclin-dependent kinase inhibitor p21 and proapoptotic genes Puma and Bax. Moreover, phosphorylated p53 transcriptionally induces most of its negative regulators, including HDM2, UBE4B, and Wip1, via negative feedback loops. Once DNA damage is resolved or p53 activity is not needed, p53 and most of its negative and positive regulators undergo dephosphorylation by Wip1. Moreover, UBE4B binds and degrades phosphorylated p53. This figure was created using BioRender.com (granted a license “Academic License Terms”, No. BH246NTRVL).

p53 is rapidly activated and stabilized through posttranslational modifications in response to multiple internal and external cellular stresses (Fig. 2). The response of activated p53 has been proven to be highly complex and cell- and context-dependent. In general, activated p53 responses is thought (i) to facilitate cell survival by activating cell cycle arrest and DNA repair programs and (ii) to promote cell death programs by triggering senescence and apoptosis pathways. The latter pathways are considered to be the major p53-induced response pathway and gold standard targets to mitigate cancer development and progression. Activated p53 can promote the programmed cell death pathway through its well-characterized channels. Moreover, p53 induces apoptosis in a transcription-dependent or -independent manner. Activated p53 transcriptionally activates numerous proapoptotic genes in intrinsic (including p53-upregulated modulator of apoptosis (PUMA), Bcl-2-associated X protein (BAX), BH3-interacting domain (BID), and NOXA), and extrinsic (including death receptors FAS and DR5) proapoptotic pathways. However, mitochondrial outer membrane permeabilization (MOMP, a hallmark of the intrinsic apoptotic pathway) is targeted by p53 in a transcription-independent manner^41^. Modified p53 proteins can translocate from the nucleus to the cytoplasm under different cellular stress conditions. In the cytoplasm, p53 can bind to the B-cell lymphoma-2 (Bcl-2) protein family, leading to MOMP and cytochrome-c release. Recently, abundant evidence demonstrated that apoptosis is not the only p53-targeted cell death program. Researchers have identified p53 as a facilitator of the death of damaged cells through ferroptosis, pyroptosis, necrosis, and autophagy^42^. Autophagy criteria have also been recently clarified and detailed. Senescence is another major outcome of p53 activation in response to dysfunctional telomeres and cellular stresses. Senescent cells are irreversible and nonproliferating living and functioning cells. Under normal conditions, cells undergo senescence in response to telomere shortening after several replication cycles. However, different cellular stresses (for instance, DNA damage and oncogenic activation) can trigger senescence in a process generally referred to as stress-induced premature senescence (SIPS)^41,43^. p53-induced SIPS is modulated through a p53 classical target: the cyclin-dependent kinase inhibitor p21. Sustained p21 induction may lead to p16^INK4A^ upregulation, which eventually activates the senescence program through the retinoblastoma pathway^44^. Moreover, p53 can directly induce senescence by stabilizing plasminogen activator inhibitor-1 (PAI-1), a marker of senescent cells^45^. Several research groups have shown that different cancer cell lines undergo senescence not apoptosis in response to ionizing irradiation^46^. The other typical p53 response to cellular stresses involves promotion and modulation of cell cycle arrest and DNA repair. It has been well documented that p53 halts cell cycle progression and induces p21 activation^47^. Once cell cycle progression is halted, p53 mediates the activation of different DNA repair mechanisms.

p53 family members p63 and p73 also respond to cellular and genomic stresses by promoting the expression of genes involved in cell cycle arrest, DNA repair, apoptosis, and autophagy^48,49^. Intriguingly, studies demonstrated that p53-induced apoptosis in response to DNA damage depends on functional p63 and p73^50^. In particular, in the combined absence of p63 and p73, cells with functioning p53 were unable to undergo apoptosis in response to DNA damage. Moreover, the transcriptional activities of p63 and p73 were essential for inducing several DNA repair genes, including BRCA2 and Rad51^48^.

The tumor suppressor p53 can promote cell death continuously in response to cellular insults; however, the question remains: what events determine the different p53-induced responses? Although many factors may impact the outcome of the p53-induced response, including the cell type, microenvironment, nature of stress, and damage severity, the answers to this intriguing question are yet to be fully elucidated. However, several proposed models may elucidate the definitive fate selectivity of the p53-induced response. One popular model suggests the dynamic behaviors of p53 in response to cellular stresses. The term dynamic behavior refers to the variations in the content level, subcellular localization, and/or PTM of a specific protein that are induced through specific stress-inducing stimuli^51,52^. In response to DNA damage, p53 and its upstream regulators/activators (such as ATM-CHK2 and ATR-CHK1), negative feedback loop molecules (such as Hdm2 and Wip1), and downstream targets (such as p21) exhibit repeated pulses/oscillations or other forms of dynamic behaviors^46,53^. The duration and intervals of the induced oscillation in p53 activity are damage- and cell type-dependent. It has been demonstrated that sustained p53 induction may promote the activation of cell death pathways. In contrast, pulsed p53 induction may facilitate cell cycle arrest and DNA repair pathways^54,55^. Using a sophisticated mathematical/computational model, Purvis J. et al.^54^ demonstrated that different p53 dynamics trigger different cellular responses. Moreover, other spatial and subsequent PTMs may dictate and facilitate the clear fate of a p53 response. One of the most canonical examples of this fate direction is mediated by the phosphorylation of p53 at S46 via homeodomain interacting protein kinase 2 (HIPK2), which directs p53 to transactivate proapoptotic genes^56,57^.

## Complex PTMs, phosphorylation and ubiquitination, govern the activity of p53 in response to DNA damage

Because p53 plays a continuous central role in a broad range of cellular activities, p53 levels, activities, and cellular localization are precisely regulated through several mechanisms, including posttranslational modifications, protein–protein interactions, and microRNAs. It has been demonstrated that maintaining the p53 basal expression level is necessary to mediate its homeostatic function. Thus, p53 is continuously turned over, which explains its short half-life of fewer than 20 min. However, once p53 is needed for its stress-induced functions, p53 can be rapidly activated and stabilized. Hence, turnover, stabilization, and other events directed by p53 are precisely regulated by a variety of posttranslational modification (PTM) mechanisms, including phosphorylation, acetylation, neddylation, SUMOylation, and ubiquitination^58^. In this regard, ~15% of the 393 amino acids in p53 are regularly modified. Most of these residues are located at the C- and N-termini of p53. It has been documented that different PTMs engage in crosstalk and interactions with each other to efficiently and precisely guide p53 activities in a context- and tissue-specific manner. Therefore, aberrant expression of PTM-mediating molecules leads to the inactivation of p53 in many cancers. Therefore, studies into targeting p53 regulators to reactivate p53 is an active research direction. Here, we discuss ubiquitination and phosphorylation and their counteracting processes, namely, deubiquitination, and dephosphorylation, respectively (Table 1).

**Table 1:** Selection of frequently reported molecules that mediate the PTMs of the p53 protein under homeostatic and stress conditions.

Protein	Type	PTM	Effect on p53	Deletion phenotype in mice
MDM2	E3 ubiquitin ligase, RING-type	Ubiquitination	Nuclear export, degradation	Embryonic lethal
Pirh2	E3 ubiquitin ligase, RING-type	Ubiquitination	Degradation	Viable
Cop1	E3 ubiquitin ligase, RING-type	Ubiquitination	Degradation	Embryonic lethal
UBE4B	E3/E4 ubiquitin ligase, U-box type	Ubiquitination	Degradation	Embryonic lethal
CHIP	E3 ubiquitin ligase, U-box type	Ubiquitination	Degradation	Viable, aging
Trim24	E3 ubiquitin ligase, RING-type	Ubiquitination	Degradation	Viable
USP7	Deubiquitinating enzyme	Deubiquitination	Stabilization, degradation	Embryonic lethal
ATM	Kinase	Phosphorylation	Stabilization	Viable, acutely radiosensitive
ATR	Kinase	Phosphorylation	Stabilization	Embryonic lethal
CHK1	Kinase	Phosphorylation	Stabilization	Embryonic lethal
CHK2	Kinase	Phosphorylation	Stabilization	Viable
DNA-PK	Kinase	Phosphorylation	Stabilization	Viable
WIP1	Phosphatase	Dephosphorylation	Destabilization	Viable, cancer resistant

## Phosphorylation of p53: the ATM-CHK2 and ATR-CHK1 axes are the dominant modulating pathways of p53 activity in response to multiple types of DNA damage

Protein kinases are the best-known DNA damage modulators. They transmit signals from a damage site to different targets (the hub of all these targets is the p53 protein) through phosphorylation. Most phosphorylation p53 events lead to its stabilization, accumulation, and translocation into the nucleus^59^. In the nucleus, p53 aggregates as tetramers (the active form of p53) and ultimately transcriptionally activates/represses its target genes. Multiple protein kinases modify numerous serine and threonine residues in p53. The most commonly phosphorylated serine/threonine residues (including S15, T18, S20, S46, and S392) are located in the N-terminal transactivation domains and C-terminus of p53^60,61^. When phosphorylated, p53 is inaccessible to negative regulators. For example, a study showed that phosphorylation of p53 at S15 in response to DNA damage promotes p53-MDM2 dissociation and leads to p53 accumulation^62^. Furthermore, phosphorylation of certain residues may lead to specific p53 physiological outcomes. For instance, phosphorylation of p53 at serine 46 by HIPK2 may lead to the transaction of proapoptotic genes, such as PUMA.

Phosphatidylinositol 3-kinase-related kinase (PIKK) members (including ATM, ATR, and DNA-PK) play leading roles in regulating, facilitating, recognizing, and amplifying the DDR multifunctional signaling pathways that modulate cell cycle arrest, DNA repair, senescence, and apoptosis^63^. Therefore, aberrations to or loss of these kinases predispose cells to genetic alterations, leading to multiple disorders, including cancer.

ATM and ATR orchestrate cell cycle arrest and DNA repair pathway signaling, an intracellular communication mechanism that has been extensively studied. This coordinated signaling is realized principally by ATM and ATR targeting of their downstream effectors, including the checkpoint kinases checkpoint kinase 2 (CHK2) and checkpoint kinase 1 (CHK1), respectively. Thus, the extensively characterized ATM-CHK2 and ATR-CHK1 pathways are activated^64^. The ATM-CHK2 pathway plays a significant role in the response to DSBs, while the ATR-CHK1 pathway is frequently activated in response to replication stalling, SSBs, and base modifications. Moreover, the ATR-CHK1 pathway is activated and necessary for DSB repair. Furthermore, ATM is activated by ATR in response to UV exposure^65^. Thus, the two pathways overlap and collaborate in response to different DNA-damaging stimuli^66,67^. CHK2 is also a Ser/Thr kinase and has been investigated in-depth since its discovery. In intact cells, CHK2 is an inactive monomer, which is swiftly phosphorylated at T68 by ATM in response to DNA damage^68,69^. Once phosphorylated, CHK2 undergoes autophosphorylation and dimerization, which leads to its full activation^70^. ATM and CHK2 target many shared and exclusive substrates that may amplify DDR signaling, further activating substrates and leading to distinct outcomes. For instance, the ATM-CHK2 pathway can halt cell cycle progression in response to DNA damage by targeting different pathways. One of the ATM-CHK2 activated pathways suppresses the cell cycle in response to DSBs after CHK2-dependent phosphorylation/inhibition of cell division cycle 25 (Cdc25A) and Cdc25C phosphatases, leading to the inhibition of cyclin-dependent kinase 2 (CdK2) and Cdk1 activity, respectively. The other pathway targeted by ATM-CHK2 is involved in the direct phosphorylation and activation of the p53 pathway, which eventually transcriptionally activates the cyclin-dependent kinase inhibitor (p21), which then negatively regulates Cdk2, 4 and 6 activity^67,71,72^. Furthermore, although the role played by ATM during the cellular response to DSBs has been the most investigated action, ATM has also been demonstrated to participate in other pathways in response to different types of lesions. Thus, loss of ATM activity leads to an inadequate response to DSBs, as highlighted in ataxia-telangiectasia syndrome (A-T). Patients with A-T present with an inherited mutated/dysfunctional ATM^73^. One of many characteristics of A-T is acute radiosensitivity, which predisposes A-T patients to malignancies (most commonly affecting the lymphoreticular system). Patients with one of several solid cancers, including breast, pancreatic, and colorectal cancers, also present with a loss of ATM expression^74–76^. CHK2 dysfunctional mutations have also been reported in other cancers, such as prostate and breast cancers^77^.

Similar to CHK2, CHK1 is a Ser/Thr kinase that is rapidly phosphorylated, by active ATR at its S345 residue, leading to its autophosphorylation and full activation^78^. Similar to activation of the ATM-CHK2 axis, activation of the ATR-CHK1 axis prevents cell cycle progression after DNA has been damaged. In addition, CHK1 phosphorylates and inactivates Cdc25A and Cdc25C through proteasomal degradation. As discussed above, ATM-CHK2 and ATR-CHK1 are the central kinases that constantly phosphorylate p53 and its negative regulators (such as MDM2, MDM4, Cop1, and Trim24) in response to different types of cellular stresses and DNA damage^79–82^. In general, phosphorylation of the negative regulators of p53 leads to their own destabilization and degradation.

Although the ATM-CHK2 and ATR-CHK1 pathways respond to distinct types of damage, their actions overlap, and their collaborative response compensates for each other in response to different DNA damaging agents. Interestingly, unpublished data from our laboratory showed that ATM is dispensable for p53 phosphorylation in response to ionizing radiation (IR). In an ATM-deficient cell line, p53 was phosphorylated at S15 and S392 in response to IR treatment (data published in a Ph.D. thesis 10.7939/R3BV7BB6R). These data emphasize the universality and powerfulness of the protein kinase ATR. Compared to ATM, ATR may modulate a higher number of signaling networks in response to DNA damage.

Interestingly, ATM phosphorylates different p63 isoforms, which leads to distinct outcomes. In particular, after phosphorylation by ATM, ΔNp63 is destabilized, facilitating the induction of proapoptotic genes^83^. In addition, ΔNp73 destabilization is crucial to cell death in response to DNA damage^84^. In contrast, in response to DNA damage, phosphorylation of TAp63 and TAp73 leads to their stabilization^85^.

Generally, the ATM-CHK2 and ATR-CHK1 pathways promote cell survival as an initial mitigation in response to DNA damage. Furthermore, loss of their activity commonly leads to cell sensitivity to radiation but does not stop DNA synthesis (replication). Thus, many cancer cells rely on DDR components, including ATM and ATR, to survive DNA-damaging events. For instance, the ATR-CHK1 pathway is frequently activated in response to replication stress, and notably, cancer cells are under high levels of replication stress. Therefore, cancer cells rely on ATR-CHK2 pathway activation to circumvent harmful threats caused by replication stress. In this sense, targeting ATM-CHK2 and ATR-CHK1 pathways and their numerous substrates/components may sensitize cancer cells to DNA-damaging agents. This strategy has been proven effective as shown by the many small-molecule inhibitors of these pathways that have been and continue to be developed (Table 2). A number of these small molecules have been entered into clinical trials and are being investigated as single or combination treatments for various cancers. For instance, AZD0156, an ATM inhibitor, is in a phase I clinical trials, where it is being administered as a single treatment or in combination with FDA-approved olaparib (a PARP inhibitor). In a lung xenograft model, AZD0156 was found to sensitize cancer cells to radiation therapy. AZD0156 significantly enhanced olaparib effects on breast, lung, and gastric cell lines in combination with olaparib^86^. AZD1390 is another ATM inhibitor currently being investigated in phase I clinical trials. AZD1390 has demonstrated high potency in preclinical experiments in which it sensitized brain cancer cells to radiotherapy^87^.

**Table 2:** Selection of drugs targeting p53-related networks in clinical trials as indicated in the ClinicalTrials.gov database.

Drug name	Target	Effect of p53	p53-dependent phenotype in tested cancer cells	Clinical stage (status)	Combination	Diseases	NCT #
APR-246	Mutant p53	Restores wild-type functions	Apoptosis^178^	Phase 2 (completed)	Azacitidine	TP53-mutant AML	NCT03931291
				Phase 3 (completed)	Azacitidine	TP53-mutant myelodysplastic syndromes	NCT03745716
				Phase 2 (completed)	PLD	High-grade serous ovarian cancer	NCT03268382
COTI-2		Restores wild-type functions	Apoptosis and senescence in HNSCC^179^	Phase 1	Cisplatin	Gynecological tumors, head and neck squamous cell carcinoma	–
Nedisertib (M3814, peposertib)	DNA-PK	Induces p53	Apoptosis in AML^180^	Phase 1 (Recruiting)	Radiation Temozolomide	Glioblastoma	NCT04555577
				Phase 1 (Recruiting)	PLD	Ovarian cancer	NCT04092270
–		–	–	Phase 1 (completed)	PLD	Advanced solid tumor	–
–		–	–	Phase 1	Enzalutamide	Prostate cancer	–
–		–	–	Phase 2	Prexasertib	Triple-negative breast cancer	–
–		–	–	Phase 1, Phase 2	PLD	Advanced malignancies	–
RG7388 (Idasanutlin)	Hdm2	Stabilizes/activates p53	Apoptosis of osteosarcoma cell line^181^	Phase 1, Phase 2 (recruiting)	Venetoclax, other chemotherapies	AML, ALL Neuroblastoma Solid tumors	–
DS-3032b (Milademetan)		Stabilizes/activates p53	Apoptosis^182^	Phase 1, Phase 2 (completed)	Cytarabine, Venetoclax	AML	–
–		Stabilizes/activates p53	Apoptosis^183^	Phase 2, Phase 3 (recruiting)	Doxorubicin	Liposarcoma	–
AMG-232 (Navtemadlin)		Stabilizes/activates p53	Apoptosis of ALL cells^184^ Cell growth inhibition and apoptosis^185^	Phase 1 (recruiting)	Cytarabine, Idarubicin hydrochloride	AML	–
APG-115		Stabilizes/activates p53	Cell cycle arrest and apoptosis in AML^186^	Phase 2 (recruiting)	–	T-prolymphocytic leukemia	–
CGM097		Stabilizes/activates p53	Cell cycle arrest and apoptosis in neuroblastoma^187^	Phase 1 (completed)	–	Solid tumors	–
–	ATM	–	–	Phase 1	Olaparib, irinotecan, fluorouracil, folinic Acid	Advanced solid tumors	–
–		–	–	Phase 1	Radiation	Brain tumor	–
–	ATR	–	–	Phase 1 (recruiting)	Irinotecan hydrochloride	Solid tumors	NCT02595931
				Phase 2 (completed)		Solid tumorsLeiomyosarcomaOsteosarcoma	NCT03718091
–		–	–	Phase 1 (recruiting)	Niraparib	Advanced solid tumors Ovarian cancer	–
–	CHK1	–	–	Phase 1 (completed)	Gemcitabine	Solid tumors	–
MK-8776 (SCH 900776)		–	–	Phase 1 (completed)	Gemcitabine	Hodgkin disease lymphoma, non-Hodgkin	NCT00779584
LY2606368 (Prexasertib)		–	–	Phase 1 (completed)	–	Advanced cancer	NCT02778126
				Phase 2 (completed)		Small-cell lung cancer	NCT02735980
				Phase 2 (completed)		Ovarian cancerBreast cancerProstate cancer	NCT02203513
SRA737		–	–	Phase 1/2 (completed)	–	Advanced solid tumors Non-Hodgkin lymphoma	NCT02797964
				Phase 1	Gemcitabine, Cisplatin	Advanced solid tumors	NCT02797977
				Phase 2 (completed)	Gemcitabine	–	–

*PLD* pegylated liposomal doxorubicin hydrochloride, *AML* acute myeloid leukemia, *ALL* acute lymphocytic leukemia, *HNSCC* head and neck squamous cell carcinoma.

Inhibition of ATR kinase activity through small-molecule inhibitors has also been applied to sensitize cancer cells to DNA-damaging agents. One of the first characterized potent ATR inhibitors was NU6027, which can sensitize breast cancer cells to DNA-damaging agents, such as cisplatin^88^. Treating acute myeloid cell lines with AZ20 (another ATR inhibitor) combined with cytarabine increased the apoptosis rate. At the same time, AZ20 and gemcitabine demonstrated dramatic growth inhibition of pancreatic cancer cell lines^89,90^. One very promising ATR inhibitor is M6620, which has been entered into phase II clinical trials. M6620sensitizes cancer cell types to various DNA-damaging agents, including radiation, cisplatin, and gemcitabine^91–95^. Very recently, RP-3500 was identified as an ATR inhibitor. It showed excellent preclinical pharmacodynamics and high potency^96^. RP-3500 is currently in phase I clinical trials as a single agent or in combination with PARP inhibitors (NCT04497116). Small-molecule inhibitors targeting the kinase activities of CHK2 and CHK1 have also been successfully developed, and some have been entered into clinical trials. Moreover, in line with CHK2-knockout mice, inhibition of CHK2 by several inhibitors (such as BML-277) led to radioresistance in the treated cells^97,98^. Despite recent discoveries, identification of CHK2 inhibitors is still an active area of research. For instance, PV1019 showed high potency and specificity in inhibiting CHK2 activity^99^. Moreover, PV1019 has been shown to sensitize U251 cells to radiation. An inhibitor that has been advanced into clinical trials is AZD7762, a dual inhibitor of CHK2 and CHK1^100^. Unfortunately, the clinical trials were precluded due to cardiac toxicity induced by AZD7762. Targeting CHK1 via inhibitors has been much more successful than targeting CHK2. Several CHK1 inhibitors have been identified and shown to exhibit high potency. These CHK1 inhibitors include PF-477736, MK-8776, and LY2606368, all of which have demonstrated promising outcomes either as a single agent or in combination with several DNA-damaging agents in preclinical experiments^101–106^. These CHK1 inhibitors and others (SRA737 and UCN-01) have been advanced into clinical trials^107,108^.

Phosphorylated and activated p53 levels must be attenuated to their preinduced levels once p53 activity is not needed. Several documented phosphatases mediate this task, of which wild-type p53-induced phosphatase 1 (Wip1, also known as PPM1D) has been extensively studied^109^. Wip1 is a crucial regulator of the DNA damage response^110^. Wip1 diminishes the DDR by dephosphorylating and inactivating upstream regulators and downstream effectors of the DDR, including H2AX, p53, ATM, ATR, MDM2, MDM4, CHK1, CHK2, p21, and p38 mitogen-activated protein kinase (MAPK)^80,111–117^. Therefore, the stressed cell bypasses cell cycle checkpoint, apoptosis, and DNA repair mechanisms. Accordingly, it is not surprising that, in response to ionizing radiation (IR), Wip1 interacts with and dephosphorylates the proapoptotic BAX protein, enabling the IR-exposed cell to escape BAX-mediated apoptosis^118^. Recently, a few negative regulators of Wip1 have been described, including miR-16 and HIPK2^119,120^. HIPK2 has been shown to physically interact with and phosphorylate Wip1, which leads to its proteasomal degradation. Thus, Wip1 is considered a universal phosphatase.

## Ubiquitination of p53: many E3/E4 ubiquitin ligases share the ultimate goal of deactivating the p53 protein

Under nonstress conditions, p53 is continuously expressed but is maintained under its inducible threshold level via constant degradation. p53 degradation is primarily induced by the well-characterized ubiquitin‒proteasome system (UPS)^121^. UPS is an enzymatic cascade involving several distinct enzymes that facilitate and mediate the sequential attachment of ubiquitin (Ub) molecules to lysine residues of a substrate. The ubiquitin enzymes include (a) E1 ubiquitin-activating enzymes, (b) E2 ubiquitin-conjugating enzymes, (c) E3 ubiquitin ligases, and (d) E4 ubiquitin chain assembly factors^122,123^. A ubiquitin-tagged substrate is recognized and destroyed by the well-characterized 26S proteasome. A brief and simple description of the ubiquitination process starts with the E1 enzyme, which recruits and activates ubiquitin molecules in an ATP-dependent manner. Subsequently, the activated Ub molecules are transferred to E2. An E3 ubiquitin ligase then mediates the attachment of Ub molecules to a specific substrate. In some cases, E4 enzymes elongate the Ub chains tagged to a substrate to form a polyubiquitinated chain. While monoubiquitination mediates several substrate outcomes, such as subcellular localization and membrane trafficking, polyubiquitinated substrates are the only form recognized by the 26S proteasome for degradation^124^.

Polyubiquitination forms chains of Ub molecules attached to their lysine residues K6, K11, K27, K29, K33, K48, and K63^124^. Chains that are linked at a K48 residue and contain more than 4 Ub molecules are predominantly recognized by the proteasome^125^. Thus, E3 and E4 ubiquitin ligases play major roles in controlling the specificity of ubiquitinated substrates and the outcomes of this process. Based on their catalytic domains, E3/E4 ubiquitin ligases are classified into the (a) RING (really interesting new gene) type, (b) U-box type, and (c) HECT (homologous to E6-AP carboxyl terminus) type. E3/E4 ubiquitin ligases with RING and U-box domains function by binding to both E2 and a substrate to directly facilitate the transfer of Ub to the substrate. On the other hand, the Ub molecule is first transferred from E2 to a HECT-type E3 ligase, and the latter then transfers Ub to the substrate. The list of identified E3 and E4 ubiquitin ligases that are involved in monoubiquitination, multiple monoubiquitination or polyubiquitination of p53 is increasing. Several lysine residues in p53 have been found to be targets for ubiquitination. Based on the required outcome of p53 ubiquitination, different E3 ubiquitin ligases are recruited to ubiquitinate specific lysine residues. The mouse double-minute two gene (MDM2) was one of the first discovered and has been among the most extensively studied E3 ubiquitin ligases that fine-tune the level and activity of p53^126^. Mouse studies revealed that deletion of *MDM2* was embryonic lethal^127^. Moreover, MDM2 is transcriptionally targeted by p53, creating an autoregulatory feedback loop^128^. Additionally, we showed that MDM2 was a transcriptional target of p73. HDM2-mediated ubiquitination of p73 led to the inhibition of its tumor suppression activities, including cell cycle arrest and apoptosis^129^.

MDM2 can mediate only the mono- or multiple-ubiquitination of p53, which leads to p53 shuttling from the nucleus to the cytoplasm^130,131^. Several negative and positive regulators impact MDM2 activity, stability, and degradation. One of the most studied MDM2 partners is MDM4 (also known as MDMX)^132^. MDM4 binds to MDM2 and stabilizes and facilitates its ubiquitin ligase activity^132,133^. Following the identification of MDM2 (also called HDM2 in humans), several other E3 ubiquitin ligases targeting p53 were gradually characterized. We previously demonstrated that a p53-induced protein with a RING-H2 domain (Pirh2) directly bound p53 and mediated its ubiquitination and degradation independent of HDM2 activity^134^. The expression of Pirh2 significantly decreased p53-mediated apoptosis and cell cycle arrest. Similar to Hdm2, Pirh2 is directly and transcriptionally activated by p53, providing a negative feedback loop. Moreover, we showed that Pirh2 bound and ubiquitinated p73, preventing its transcriptional effects.

Tripartite-motif-containing protein 24 (Trim24) is another E3 ubiquitin ligase with a RING domain that directly binds and ubiquitinates p53^135^. Aberrant expression of Trim24 is evident in multiple cancers, including breast cancer^136^. Another interesting E3 ubiquitin ligase is constitutive photomorphogenesis protein 1 (Cop1)^137^. Cop1 also features a RING domain. Similar to MDM2, Cop1 is a transcriptional target of p53. The carboxy terminus of Hsp70-interacting protein (CHIP) is another crucial E3 ubiquitin ligase domain that targets p53^138^. CHIP contains a U-box domain that enables CHIP polyubiquitination of its substrates. CHIP mediates the proteasomal degradation of mutant p53^139^. Recently, our group showed that p63 isoforms were direct substrates of CHIP^140^. CHIP physically bound and ubiquitinated TAp63 and ΔNp63, which led to their proteasomal degradation. Interestingly, we found that heat shock protein 70 (Hsp70) was a molecular switch that guided CHIP-mediated ubiquitination of p63 isoforms. The absence of Hsp70 led to increased ubiquitination and degradation of ΔNp63 by CHIP.

We demonstrated that ubiquitination factor E4B (UBE4B in humans, Ufd-2 in yeast, Ufd2a and Ube4b in mouse) is required for Hdm2-mediated polyubiquitination and degradation of p53^141^. Thus, in unstressed cells, unphosphorylated p53 (inactive) is maintained in check by the cooperative activity of Hdm2 and UBE4B and other E3/E4 ubiquitin ligases. Previous studies have shown that phosphorylated p53 (active) is not affected by E3/E4 ubiquitin ligases, including Hdm2. Thus, dephosphorylation of p53 is a prerequisite for its ubiquitination. However, it has been reported that CARPs (caspase 8/10-associated RING proteins) bind and degrade phosphorylated p53 at S20^142^. Our group showed that, in response to IR, UBE4B bound and degraded phosphorylated p53 at Serine 15 and 392 residues. Hence, a limited number of p53-related E3//E4 ubiquitin ligases are capable of modulating the activity of phosphorylated p53^143^. Interestingly, a research group found that inhibition of UBE4B activity (probably via phosphorylation) led to cell cycle arrest at G2 and that these cells did not advance to mitosis^144^. They identified phosphorylated UBE4B mainly on the basis of the molecular weight of the different UBE4B proteins detected. Moreover, using *Caenorhabditis elegans* germ cells, Ackermann L. et al. showed that Ufd-2 formed foci in DNA-damaged sites, which was crucial to facilitating the activation of proapoptotic pathways induced by IR treatment^145^. Furthermore, they established that Ufd-2 activity was required for the timely release of RAD51 from a damage site; without RAD51 release, DNA repair was inefficient. The authors concluded that Ufd-2 was crucial to efficiently facilitate the coordination of DNA repair and apoptotic networks. Importantly, Cdc48, a cell cycle-promoting molecule, is a Ufd-2 binding partner, and the interaction of these proteins is indispensable for substrate degradation^146^. Similarly, the Ufd-2–RAD23 interaction is essential for substrate degradation. Interestingly, the UBE4B homolog UBE4A has recently been demonstrated to be recruited to DNA damage sites^147^. At this foci, recruitment of UBE4A is required for the timely recruitment of receptor-associated protein 80 (RAP80) and BRCA1, which are needed to repair DSBs efficiently. Similarly, UBE4B recruitment has been identified in HCT116 cells responding to DNA damage^148^. We also found that UBE4B is a direct target of the microRNA 1301 (miR-1301)^149^. We and others have documented the role played by miR-1301 in mediating p53 stabilization and repression of cell migration and invasion^149,150^.

In summary, our studies and those of others indicated that, although it is not fully understood, UBE4B may modulate different networks needed to facilitate DNA repair, cell cycle arrest, and cell fate in response to DNA damage in a p53-dependent manner. E3/E4 ubiquitin ligases play central roles in regulating the tumor suppressor p53 and its family members in response to DNA damage. Targeting many of these ligases sensitizes cancer cells to DNA-damaging agents.

The ubiquitination of p53 is reversible through the action of deubiquitinating enzymes (DUBs), which remove Ub molecules and chains. DUBs also regulate the stability and activity of p53 by targeting its regulators^151^. DUBs constitute a large family of proteins that can be categorized into several subgroups, the largest of which is the ubiquitin-specific protease (USP) subfamily. One of the USP proteins that impacted the activity of p53 and stability to be discovered was USP7 [also known as herpes virus-associated ubiquitin-specific protease (HAUSP)]^152^. It has been shown that USP7 deubiquitinates p53, leading to p53 stabilization^153^. However, USP7 inhibition also leads to the stabilization of p53^154^. Notably, USP7 has been shown to exhibit a higher binding affinity for MDM2, which it deubiquitinates, increasing its stability and facilitating MDM2-dependent degradation of p53^154,155^. Furthermore, MDM4 is a direct substrate of USP7^156^. In addition, there are many other USP family members (such as USP2a, USP2, and USP4) that directly facilitate the stabilization of negative regulators of p53^157–159^. For instance, similar to USP7, USP2a inhibits the ubiquitination of MDM2 and MDM4, leading to their stabilization and facilitating their interactions with p53^157,160^. On the other hand, USP10, USP11, USP24, and USP29 have been reported to directly impact p53 activity by deubiquitinating and stabilizing it^161–164^. The ever-increasing list of molecules/players in this process is clear evidence that the tumor suppressor p53 is essential to many biological functions in different tissues and contexts.

## Reactivation of p53 as a targeted therapy

Reactivation of the p53 pathway has been a target of anticancer therapy for decades. Several small-molecule inhibitors targeting negative regulators of p53 activity have been widely applied in combination with radio- and chemotherapies to obtain the optimum activation of the p53 pathway in different cancers with wild-type p53. Many of these inhibitors are currently in clinical trials (Table 2). Among these leading inhibitors, Nutlins are MDM2 inhibitors^165,166^. Nutlins simply interact with p53 at the MDM2-binding site with higher potency than MDM2 and thus mediate the stability of p53. Many other inhibitors that target the MDM2–p53 interaction have been identified^167^. Another means of p53 reactivation as a gene therapy is direct introduction of functional wild-type p53 into cancer cells^168^.

Furthermore, missense mutations are frequently transcribed and translated into stable full-length mutant forms of p53. Moreover, small molecules that can refold certain p53 mutants into a the wild-type conformation have been developed^169^. For instance, PRIMA-1 is a small-molecule inhibitor that can restore the normal conformation and activities of p53 by binding to mutant p53 (R273H and R157H)^170–172^.

## Conclusions and perspectives

DNA-damaging agents, including radiotherapy, are widely used in many clinical settings to kill cancer cells and/or slow their proliferation. Furthermore, these agents are very useful in mitigating cancer-related symptoms in advanced and inoperable cancers. In fact, more than one-half of all cancer patients receive radiotherapy as part of their treatment regimen^173^.

The tumor suppressor p53 is at the hub of the DNA damage response. PTMs primarily regulate p53 activity in response to DNA damage. Targeting PTM-induced regulators of p53 has been proven in bench and clinical settings to sensitize cancer cells to DNA-damaging agents. Although many drugs targeting p53 regulators have been developed, resistance and recurrence following treatment are not uncommon. Thus, new treatments/targets are urgently needed.

We extensively studied the tumor suppressor p53, its family, and its regulatory network in cancer^129,140,141,143,149,174–176^. We recently showed that UBE4B independently and negatively regulated phosphorylated p53 in response to ionizing radiation, emphasizing that UBE4B may play an important role during the active cellular response to DNA damage^143^. Moreover, several other studies have reported the role played by UBE4B in response to DNA damage^145,148,177^. However, the mechanism governing UBE4B regulation in response to DNA damage in cancer is still largely unknown. Moreover, in addition to p53, what are other DDR molecules are regulated by UBE4B? Is UBE4B involved in DNA repair mechanisms? These are among several questions currently and actively under investigation in our laboratory, and other groups are highly encouraged to investigate them. This is a novel opportunity to identify and develop an effective sensitizing agent to improve cancer treatment.

Ultimately, many researchers worldwide are constantly working to identify new molecules that are involved in the DNA damage response and function with DNA repair machinery, which may lead to the development of novel therapeutic strategies to fight cancers.
